# Genetic Differentiation and Evolutionary Adaptation in *Cryptomeria japonica*

**DOI:** 10.1534/g3.114.013896

**Published:** 2014-10-14

**Authors:** Yoshihiko Tsumura, Kentaro Uchiyama, Yoshinari Moriguchi, Megumi K. Kimura, Saneyoshi Ueno, Tokuko Ujino-Ihara

**Affiliations:** * Department of Forest Genetics, Forestry and Forest Products Research Institute, Tsukuba Ibaraki 305-8687, Japan; † Graduate School of Science and Technology, Niigata University, 8050, Igarashi 2-Nocho, Nishi-ku Niigata 950-2181, Japan

**Keywords:** LD, conifer, local adaptation, outlier locus, selection

## Abstract

Local adaptation of plant species is a central issue for survival during global climate change, especially for long-lived forest trees, with their lengthy regeneration time and spatially limited gene flow. Identification of loci and/or genomic regions associated with local adaptation is necessary for knowledge of both evolution and molecular breeding for climate change. *Cryptomeria japonica* is an important species for forestry in Japan; it has a broad natural distribution and can survive in a range of different environments. The genetic structure of 14 natural populations of this species was investigated using 3930 SNP markers. Populations on the Pacific Ocean side of Japan are clearly different from those on the Japan Sea side, as discussed in previous studies. Structure analysis and population network trees show that peripheral populations, including the most northerly and southerly ones, have unique features. We found that the genetic differentiation coefficient is low, *F*_ST_ = 0.05, although it must account for the presence of important genes associated with adaptation to specific environments. In total, 208 outlier loci were detected, of which 43 were associated with environmental variables. Four clumped regions of outlier loci were detected in the genome by linkage analysis. Linkage disequilibrium (LD) was quite high in these clumps of outlier loci, which were found in linkage groups (LGs) 2, 7, 10, and 11, especially between populations of two varieties, and when interchromosomal LD was also detected. The LG7 region is characteristic of the Yakushima population, which is a large, isolated, peripheral population occupying a specific environment resulting from isolation combined with volcanic activity in the region. The detected LD may provide strong evidence for selection between varieties.

Natural selection is the mechanism that drives the evolution of organisms, leading to individuals that are better adapted to a new environment than the original population (Endler 1986; Linhart and Grant 1996). The fitness of such adapted individuals is higher than the original organisms that spread into the new habitat and, from these, a new species may evolve (Holt and Gomulkiewicz 1997). The genetic mechanism associated with local adaptation has been studied in model plant species such as *Arabidopsis thaliana* (Fournier-Level *et al.* 2011). In long-lived plant species, however, there are few studies of adaptive mechanisms, probably because of the difficulty in evaluating phenotypes over long time periods in the field and the limited genomic information available.

Forest trees have become adapted to specific environments over many generations as the natural distribution has shifted and the population size has changed in response to past global climate change (González-Martínez *et al.* 2006; Savolainen *et al.* 2007; Neale and Ingvarsson 2008; Neale and Kremer 2011). Such climate change may result in the evolution of new species because selection pressure is different depending on climatic conditions, for example, during glacial and interglacial periods. During the past one million years, glaciation cycles of 100,000 years duration have prevailed (Howard 1997); this means that the average temperature and precipitation have fluctuated drastically between glacial and interglacial periods every 100,000 years. During glacial periods, small and isolated populations may be left behind within environmental refugia, especially in northern parts of a species’ range. Population size would be reduced under severe climatic conditions, and only resistant individuals would survive. Subsequently, offspring of the survivors would colonize out from any refugia during the interglacial period, and genetic differentiation between the isolated population and other populations is likely to have increased during their separation. Repeated glaciations would increase such genetic differentiation and drive the evolution of adaptations to survive severe conditions just like allopatric speciation.

Linkage disequilibrium (LD) throughout the genome reflects the population history, the breeding system, and the pattern of geographic subdivision, whereas LD in each genomic region reflects the history of natural selection, gene conversion, mutation, and other forces that cause gene-frequency evolution (Slatkin 2008). If we observe LD carefully in long-lived plant species, we can detect the genetic signature associated with local adaptation, even in forest tree species, because the history of the organism is recorded in the genome.

*Cryptomeria japonica* is an allogamous coniferous species that relies on wind-mediated pollen and seed dispersal. Modern natural forests of the species are distributed across various environments in the Japanese Archipelago, from Aomori Prefecture (40° 44′ N) to Yakushima Island (30° 15′ N) (Hayashi 1951). However, its distribution is discontinuous and scattered; it occupies small, restricted areas as a result of having been extensively exploited by humans over the past 1000 years (Ohba 1993). The geographical variation between natural forests of *C. japonica* has been investigated, focusing on morphological traits (needle length, needle curvature, and other features) (Murai 1947), diterpene components (Yasue *et al.* 1987), and reproductive system (Kimura *et al.* 2013). The results of these studies suggest that there are two main lines: ura-sugi (*C. japonica* var. *radicans*, found near the Sea of Japan) and omote-sugi (*C. japonica*, found near the Pacific Ocean). The ura-sugi variety has slender branchlets with soft leaves, whereas the omote-sugi variety has rough branchlets with hard leaves (Yamazaki 1995). Previous studies using 148 cleaved amplified polymorphic sequence (CAPS) loci and 1026 single nucleotide polymorphisms (SNPs) have revealed genetic differentiation between the two varieties, and four and 14 outlier loci have been identified as potential local adaptation genes, respectively (Tsumura *et al.* 2007, 2012); these may be associated with genetic differentiation of the varieties.

A high-throughput SNP genotyping system has been developed; using this, tens of thousands of genotypes can be obtained in only a few days. Genome scanning based on a large number of SNPs can result in an accurate evaluation of the genetic diversity and structure of natural populations and facilitates the detection of candidate loci associated with economically important traits and adaptive genes for specific environments (Vasemägi and Primmer 2005; Namroud *et al.* 2008; Holliday *et al.* 2010). This method may allow us to detect loci associated with adaptations that would be valuable for surviving climate change.

In this study, we focus on adaptive genes linked to past climate changes. We investigate the current genetic structure of natural populations of *C. japonica* using several thousand SNPs and characterize the outlier loci using linkage mapping and linkage disequilibrium methods. Then, we discuss their relationship to current genetic structure and how this species has adapted to the different climates experienced on the Japan Sea side and the Pacific Ocean side of the country.

## Materials and Methods

### Investigated populations

**Figure 1:**
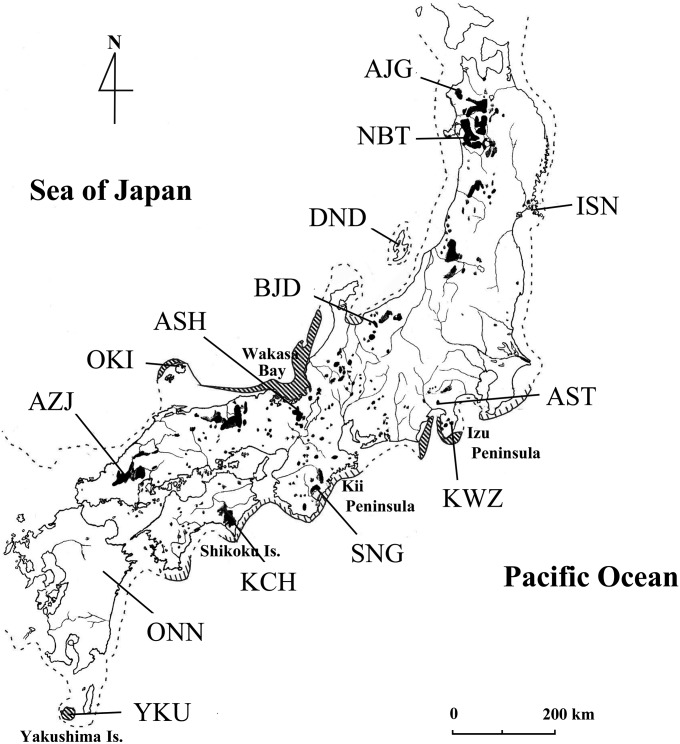
Natural distribution of *Cryptomeria japonica* in Japan (shaded areas) (Hayashi 1951) and the locations of the 14 natural populations surveyed in this study. The dotted line indicates the coastline approximately 18,000 years ago. Areas shaded in bold or within thin diagonal lines indicate established refugia (Izu Peninsula, Wakasa Bay, Oki Island, and Yakushima Island) and probable refugia, respectively, at that time (Tsukada 1986).

We examined 14 populations comprising 186 individuals that we used in a previous study (Tsumura *et al.* 2012): seven from the Japan Sea side of Japan and seven from the Pacific Ocean side (Table 1, Figure 1). All trees sampled were growing in national forests that are *in situ* gene-conservation forests in Japan. The locations of the sampled populations covered most of the natural distribution of *C. japonica*. In addition, because *C. japonica* has been widely planted since 1945 in Japan, we only sampled relatively large, old trees that were presumed to predate the widespread planting programs.

**Table 1:** Locations for 14 investigated populations of *C. japonica* and their current and last glacial period environment variables

Population	Abbrev	Lat	Long	Alt (m)	No.	Annual Mean Temp (°C)	Max Temp of Warmest Month (°C)	Min Temp of Coldest Month (°C)	Annual Precip (mm)	Deepest Snow (cm)	Annual Mean Temp in LGM (°C)	Max Temp of Warmest Month in LGM (°C)	Min Temp of Coldest Month in LGM (°C)	Annual Precip in LGM (mm)
Ajigasawa	AJG	40.6756	140.2053	319	13	9.4	26.5	−5.4	1447	70	8.5	27.3	−8.3	1304
Nibetsu	NBT	39.8061	140.2600	315	15	9.8	27.8	−5.6	1683	37	8.9	28.6	−8.5	1554
Ishinomaki	ISN	38.3286	141.4919	195	13	11.3	26.7	−3.2	1225	7	10.4	27.4	−5.6	1099
Donden	DND	38.1397	138.3833	725	14	8.4	25.3	−6.1	1924	99	7.6	26.1	−8.5	1757
Bijodaira	BJD	36.5761	137.4589	628	18	8.7	26.6	−7.7	1669	171	8.0	28.6	−9.7	1520
Ashitaka	AST	35.2322	138.8361	690	9	11.7	27.3	−4.8	2044	37	11.0	29.1	−6.8	1923
Kawazu	KWZ	34.8314	139.0000	664	16	12.6	26.4	−1.4	2327	1	11.8	27.8	−3.4	2213
Ashu	ASH	35.3078	135.7739	802	13	11.2	27.6	−3.5	1971	55	10.5	29.4	−5.6	1839
Oki	OKI	36.2683	133.3292	363	17	12.6	28.2	−1.2	1908	29	11.5	28.8	−4.1	1731
Azouji	AZJ	34.4820	131.9634	1112	8	8.6	23.9	−6.0	2245	43	7.6	25.1	−8.6	2085
Shingu	SNG	33.8900	135.7100	592	14	13.9	27.9	−0.1	2600	2	13.1	29.1	−2.2	2472
Kochi	KCH	33.5928	134.0958	615	6	12.8	26.4	−0.9	2272	2	11.9	27.2	−3.2	2134
Oninome	ONN	32.6984	131.5182	1170	4	9.4	22.9	−5.3	3227	2	8.4	23.6	−7.7	3076
Yakushima	YKU	30.3035	130.5731	1071	15	13.8	24.5	2.3	3411	0	12.7	25.0	0.3	3321

Abbrev, abbreviation; Lat, latitude; Long, longitude; Alt, altitude; Temp, temperature; Max, maximum; Min, minimum; Precip, precipitation.

### SNP genotyping

The identification of SNPs was performed previously by resequencing 5170 unique EST contigs in a discovery panel of four *C. japonica* individuals collected from a range-wide sample of trees (Uchiyama *et al.* 2012). The SNPs were also identified from transcriptome assembly of seedlings of 10 trees, range-wide, by a next-generation sequencer (Ueno *et al.* 2012; Uchiyama *et al.* 2014). All detected SNPs were EST-based coding regions. Multiplexed genotyping of SNP markers for natural populations was performed using Illumina’s four set of 1536-plex (6144 SNPs) GoldenGate array according to the protocol recommended by the manufacturer (Uchiyama *et al.* 2014). Only SNPs with Illumina design scores more than 0.6 were used. When multiple SNPs were available within the same sequence, a single highly polymorphic SNP was selected as the target for that sequence. The quality of the GoldenGate genotype scores for individual SNPs was assessed based on their GenTrain cluster and GenCall genotype scores in GenomeStudio (Illumina Inc., San Diego, California). A minimum GenCall50 (GC50) score of 0.25 was chosen as the threshold for the inclusion of SNP loci in the final data set, and genotypic clusters were edited manually when necessary. In the present study, this threshold corresponded to SNPs with accurate scoring for at least 95% of individuals, with most successful SNPs scored for more than 99% of individuals analyzed.

### Genetic structure

To evaluate the within-population variation, we used the proportion of polymorphic loci (*Pl*) at the 95% probability level, the unbiased heterozygosity (*H*_e_) (Nei 1978), and the allelic richness (*R*_s_) calculated from the allele frequencies of all loci analyzed. Allelic richness was determined using the method of El Mousadik and Petit (1996) with a sample size of 6. The fixation indices, *F*_IS_ = 1 − *H*_o_ /*H*_e_, for polymorphic loci and their averages over all loci were determined to compare the observed genotype frequencies with expectations based on Hardy-Weinberg equilibrium (Wright 1922; Nei 1977; Nei and Chesser 1983). Deviations from such expectations were analyzed using Fisher’s exact test. Coefficients of gene differentiation, *F*_ST_, between populations were calculated to determine how gene diversity was partitioned at each level (Wright 1978). These analyses were conducted using the FSTAT software package version 2.9.4 (Goudet 2003) and GenAlEx version 6.5 (Peakall and Smouse 2012). To determine any population structure and infer the most appropriate number of subpopulations (*K*) for interpreting the data without prior information about the number of locations at which the populations were sampled, we used the F-model of the Bayesian clustering approach, STRUCTURE, proposed by Pritchard *et al.* (2000). Ten independent runs with *K* values ranging from 1 to 10 were performed using 2×10^6^ MCMC (Markov Chain Monte Carlo) sampling after a burn-in period of 50,000 iterations. The posterior probability was then calculated for each value of *K* using the estimated log-likelihood of *K* to identify the optimal value. The optimal value of *K* was the one at which the log likelihood of the data, ln *P*(*X*|*K*) (Pritchard *et al.* 2000), or delta *K*, the rate of change of ln *P*(*X*|*K*) between successive *K* values (Evanno *et al.* 2005), was maximal. To examine the genetic differentiation between two groups representing the two varieties, *C. japonica* (omote-sugi) and *C. japonica* var. *radicans* (ura-sugi), we performed a hierarchical analysis of molecular variance (AMOVA) (Excoffier *et al.* 1992) in which the significance levels for variance components were tested using permutations.

We performed a phylogeographic analysis using the Neighbor-net method (Bryant and Moulton 2004) implemented in SplitsTree4 (Huson and Bryant 2006) based on the pairwise *F*_ST_ between populations to reveal more details of the evolutionary relationship between populations, for example, hybridization between populations.

#### Methods for detecting candidate loci for divergence:

We used a number of approaches to allow us to detect signs of natural selection and to identify, tentatively, the environmental parameters associated with the corresponding selective pressures acting on *C. japonica* populations. We compared the distributions of the *F*_ST_ values over all loci to their expected distributions under the assumption of neutrality. Beaumont and Nichols (1996) have shown that the distribution of *F*_ST_ as a function of heterozygosity in the context of an island model is quite robust, *i.e.*, it is insensitive to variations in factors such as population structure, demographic structure, and mutation level. We therefore used this method to identify markers that deviated from the null hypothesis of neutral evolution. All calculations for identifying potential non-neutral genes in the populations studied were performed using the FDIST2 approach of Beaumont and Nichols (1996) implemented in LOSITAN (Antao *et al.* 2008) and Arlequin version 3.11 (Excoffier and Lischer 2010). The Arlequin program allowed us to conduct hierarchical analysis using two genetic lines, here the ura-sugi and omote-sugi varieties. In the analysis using FDIST2, we identified the outlier loci using not only all 14 populations but also each type of population, *i.e.*, the ura-sugi populations and the omote-sugi populations.

An alternative method for identifying candidate loci is BayeScan version 2.1, which uses a Bayesian method to estimate a posterior probability for each locus directly and a reversible-jump MCMC approach to selection (Foll and Gaggiotti 2008). The main advantage of BayeScan is that it estimates population-specific *F*_ST_ coefficients and therefore accommodates differences in demographic history and the extent of genetic drift between populations. This method is an extension of that proposed by Beaumont and Balding (2004), and it is based on a logistic regression model that decomposes genetic variation into population and locus-specific effects. Preliminary tests were conducted using a burn-in of 10,000 iterations, a thinning interval of 50, and a sample size of 10,000 (Foll and Gaggiotti 2008). Four independent runs were performed for each of the two datasets to account for the consistency of the detected outliers. The loci were ranked according to their estimated posterior probability and all loci with a value more than 0.990 were retained as outliers. This corresponds to a log_10_ Bayes factor of more than 2, making it possible to be confident in the validity of the model.

#### Environmental data and the association with genotypes:

Environmental data regarding the area of origin of each population, including longitude, latitude, altitude, and 19 bioclimatic variables over 50 years (1950–2000), were obtained from WorldClim (Hijmans *et al.* 2005). Past climate data for the interglacial period (LIG) and last glacial maximum based on the MIROC model were obtained from the Paleoclimate Modeling Intercomparison Project Phase II (http://pmip2.lsce.ipsl.fr/). Some of the climate variables and geography such as longitude, latitude, and altitude are correlated with each other, as shown by Tsumura *et al.* (2012); therefore, principal component analysis (PCA) was used to reduce dimensionality for the climate variables and geography. Principal components (PCs) with eigenvalues greater than 1.0 were used for further analysis and PC scores were used for association analysis. All analyses were performed in R 2.12.1 (R Core Development Team 2010) using the prcomp function in the statistics package (http://cran.r-project.org/).

To calculate correlations between SNP allele frequencies and current and past climate variables, we used the Bayesian linear model method of Coop *et al.* (2010), which controls for population history by incorporating a covariance matrix of populations and accounts for differences in sample size between populations. Using the full set of SNPs, we estimated a covariance matrix of allele frequencies across populations. This matrix was used as the basis of the null model for the transformed allele frequencies at each SNP to be tested. For each tested SNP, the method generates a Bayes factor as a measure of the support for the alternative model relative to the null model in which the transformed population allele frequency distribution is dependent on population structure alone. The significance threshold for ranked Bayes factors was set to 2.0 and the environmental variables considered were latitude, longitude, and 19 climate variables—current and past based on a model with an interglacial and two LGMs—which were transformed to PC scores as described above. Putative functions for the detected outlier loci were identified by performing BLASTx searches against the NR data in the NCBI database using Blast2GO (Conesa *et al.* 2005) and the *Arabidopsis* genome annotation (TAIR10) (http://www.arabidopsis.org/).

#### Mapping the outlier loci on the linkage map and linkage disequilibrium:

The YI pedigree used to determine the positions of the outlier loci of *C. japonica* has previously been used to construct a linkage map in which a total of 1261 markers were assigned to 11 large linkage groups, giving a total map length of 1405.2 cM (Tani *et al.* 2003, Moriguchi *et al.* 2012). The mapped population was genotyped using Illumina’s 1536-plex GoldenGate array, as discussed above. All linkage analyses and map estimations were performed using the JoinMap v4.1 software package (Van Ooijen and Voorrips 2001). During map construction, markers were assigned to tentative linkage groups by comparing the linkages formed at logarithm of odds (LOD) thresholds ranging from 3.0 to 9.0, increasing in increments of 1.0. Finally, the markers were ordered at the LOD threshold of 8.0. Map distances were calculated using the Kosambi mapping function (Kosambi 1944).

Coefficients of linkage disequilibrium were calculated as described by Weir (1996) using squared allele-frequency correlations (*r*^2^) for pairs of loci on the basis of genotype data from the investigated populations. The differences from equilibrium were verified by chi-squared tests with a false discovery rate of 0.01 (Weir 1996). These analyses were performed using the R-package “genetics” (http://cran.r-project.org/web/packages/genetics/).

Assuming a random distribution of markers, if the genome was divided into N intervals, then the number of markers per interval would follow a Poisson distribution. To determine whether outlier loci were randomly distributed, every linkage group was divided into 15-, 20-, 25-, and 50-cM intervals. We used the chi-square test to compare the actual distribution of outlier loci to that expected for a Poisson distribution, as described by Kang *et al.* (2010).

## Results

### SNP genotyping

Of the 6144 SNPs, 3934 (64.0%) yielded data that met our quality thresholds according to the GoldenGate genotyping system (Uchiyama *et al.* 2012). The median GC_50_ score across all usable SNPs was 0.80, with an average call rate of 99.4%. Of those 3934 SNPs, four were monomorphic and were therefore discarded. The remaining 3930 SNPs were used in the subsequent analyses. Only 216 SNPs (5.50%, *P* < 0.05) differed significantly from Hardy-Weinberg equilibrium after Bonferroni correction.

### Genetic structure

**Figure 2:**
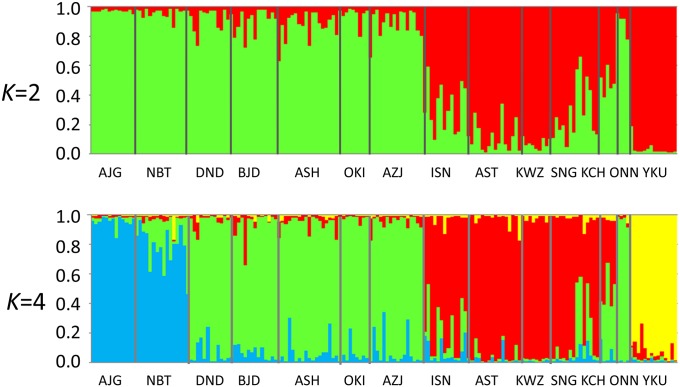
Genetic relationships among the 14 populations surveyed using STRUCTURE (Pritchard *et al.* 2000) based on 3930 SNPs. The models with *K* = 2 and *K* = 4 were optimal based on the delta K value and the highest log-likelihood value, respectively.

The *Pl* values for the SNPs ranged from 0.705 to 0.945, with an average of 0.898. The *R*_s_ values also varied, from 1.542 to 1.610, with an average of 1.591, and the *H*_e_ values ranged from 0.301 to 0.339, with an average of 0.328 (Table 2). In contrast to findings from previous studies using CAPS and SSR, these parameters do not reveal any clear geographical trends (Tsumura *et al.* 2007; Takahashi *et al.* 2005), as reported by Tsumura *et al.* (2012) using 1026 SNPs. The average *F*_IS_ value for all but 47 loci was not significantly different from expectations under Hardy-Weinberg equilibrium (*P* < 0.01). The overall genetic differentiation among populations at the 3930 loci was low (*F*_ST_ = 0.0506). We also conducted an AMOVA to determine the variation within and among groups (the two varieties) and populations, and to test the significance of the among-population variation. In this analysis, the ONN population was included in the ura-sugi variety because the result of STRUCURE analysis and the phylogenetic network clearly indicated that this was where the population belonged. Therefore, there were eight ura-sugi and six omote-sugi populations. The variation among populations was 5.06% and was highly significant (*P* < 0.001), and the proportions of variance among varieties and among populations within varieties were also significant, *i.e.*, 1.98% and 3.05%, respectively (Table 3). Bayesian clustering of the information from the 3930 loci demonstrated that the models with *K* = 2 and *K* = 4 both provided satisfactory explanations of the observed data, based on the delta *K* and the highest log-likelihood values, respectively (Figure 2). Both of the bar plots for *K* = 2 and *K* = 4 revealed clear differences between populations of the two varieties with the exception of the ONN population (Figure 2), as found in a previous study (Tsumura *et al.* 2012). In addition, the bar plot for *K* = 4 separated the populations within each variety. The ura-sugi populations were divided into two groups, with the first consisting of the most northern population and the second containing the others. The omote-sugi populations were divided into two major groups, namely YKU and the others.

**Table 2:** Genetic diversity of 14 investigated populations of *C. japonica* based on 3930 SNPs

Population	N	*Ho*	*He*	*F*_IS_	*Pl*	*Rs*	No. of Private Alleles
AJG	13	0.326	0.324	−0.008	0.901	1.583	3
NBT	15	0.324	0.330	0.019	0.922	1.593	2
ISN	13	0.329	0.333	0.014	0.915	1.599	0
DND	14	0.328	0.332	0.012	0.924	1.598	2
BJD	18	0.333	0.335	0.006	0.945	1.603	2
AST	9	0.334	0.332	−0.007	0.884	1.597	0
KWZ	16	0.330	0.333	0.009	0.934	1.599	4
ASH	13	0.331	0.338	0.019	0.940	1.608	2
OKI	17	0.325	0.333	0.024	0.940	1.599	1
AZJ	8	0.339	0.332	−0.023	0.879	1.598	2
SNG	14	0.327	0.335	0.023	0.932	1.603	2
KCH	6	0.325	0.339	0.045	0.858	1.610	2
ONN	4	0.303	0.301	−0.010	0.705	1.542	0
YKU	15	0.299	0.303	0.013	0.898	1.547	7
Mean	12.5	0.325	0.328	0.010	0.898	1.591	2.1

n, number of investigated individuals; *H*o, the observed number of heterozygosity; *H*e, the expected number of heterozygosity; *F*_IS_, fixation idenx; *Pl*, the number of polymorphic loci; *Rs*, allelic richness.

**Table 3:** Results of analysis of molecular variance

Source of Variation	d.f.	Sum of Squares	Variance Components	Percentage of Variation
Among groups	1	3578.128	13.40222	1.98
Among populations within groups	12	14042.649	20.61734	3.08
Within populations	346	222325.545	642.55938	94.94

Two varieties, *C. japonica* and *C. japonica* var. *radicans*, are used as different groups. ISN, AST, KWZ, SNG, KCH, and YKU are included into *C. japonica* (omote-sugi). The others, AJG, NBT, DND, BJD, ASH, OKI, AZJ, and ONN, are included in *C. japonica* var. *radicans* (ura-sugi).

**Figure 3:**
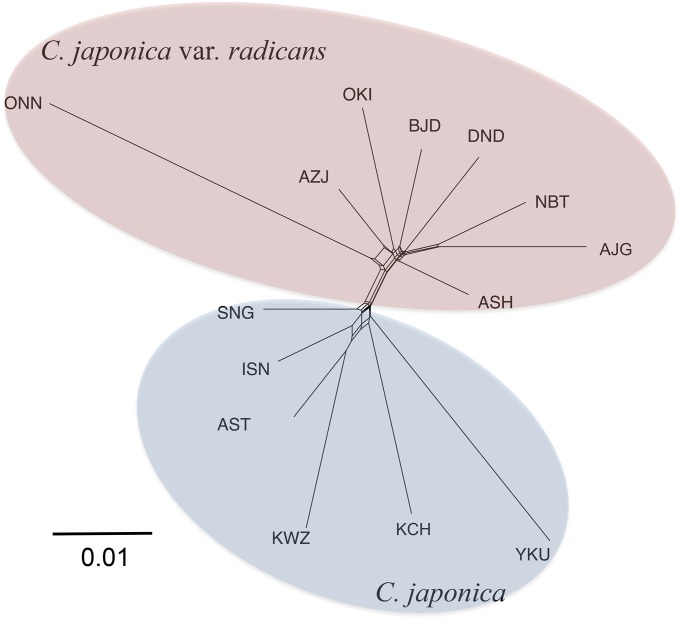
A population network based on pair-wise *F*_ST_ values between populations constructed by the Neighbor-net.

The network tree constructed using Neighbor-net indicated that the two varieties were clearly divided, and that the ONN population on Kyushu Island belonged to the ura-sugi variety, which was closed and introgressive into the neighboring AZJ population (Figure 3). The SNG population of omote-sugi that was geographically closest to the ura-sugi variety populations seemed to subject to introgression from the ura-sugi variety. The branch length of the ONN population and the YKU population suggested long-term isolation or a bottleneck.

### Outlier loci

We identified 407 loci lying outside the 99% confidence interval (C.I.), of which 199 were above the C.I., suggesting positive selection using the *F*_ST_-based method (FDIST2) (Supporting Information, Table S1). BLASTx searches identified putative functions or annotations for 178 of these loci (85.6%) (Table S1). The method of considering of the hierarchical structure, *i.e.*, the ura-sugi and omote-sugi varieties, using Arlequin identified 229 loci, of which 131 were above the C.I. (Table S2).

**Figure 4:**
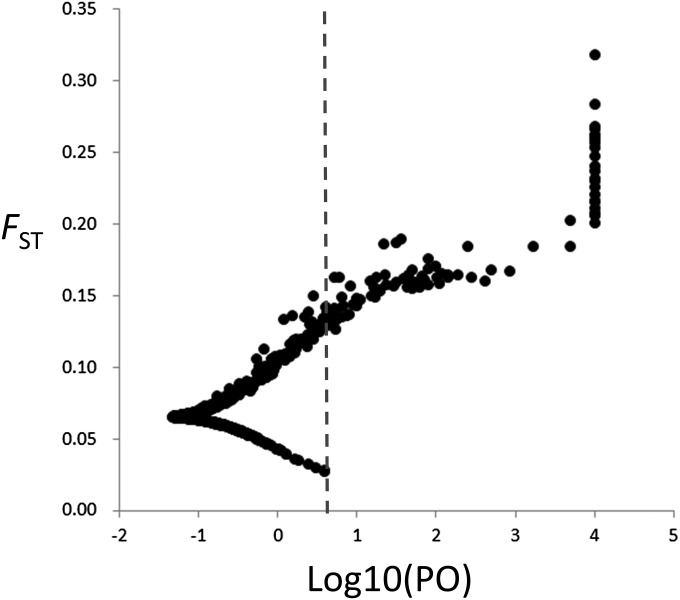
Plot of *F*_ST_ values and Bayes factors (log_10_) for 3930 loci identified using the BayeScan outlier test. Dashed lines indicate the log_10_ of the Bayes factor threshold that provides “decisive” evidence for selection corresponding to a posterior probability of 0.99.

Using the Bayesian method implemented in BayeScan, we identified 79 loci as outliers with a log_10_ Bayes factor above 1 after correction using a false discovery rate of 0.05. All but eight of these loci were also detected by the *F*_ST_-based method (Table S1, Figure S1). Of these outliers, 42 had log_10_ Bayes factor values above 2 (Figure 4); 33 of these 42 were also identified as outliers by the *F*_ST_-based method (Table S1). Thirty-five of the 42 loci had log_10_ Bayes factors above 3, corresponding to a posterior probability of locus effects above 0.999; 27 had a log_10_ Bayes factor of more than 5, corresponding to a posterior probability of 1.000. BLASTx searches identified putative functions for 36 of the 42 loci with log_10_ Bayes factors above 2 (85.7%) (Table S1).

Within-variety *F*_ST_ analysis identified 221 outlier loci. Only four of the identified outlier loci were detected in both the omote-sugi and ura-sugi populations; these were estSNPg00691, estSNPg02482, SNPg01839, and SNPg03850 (Table S2). Using the Bayesian method, 38 loci were identified in either the omote-sugi or ura-sugi populations, but no common outlier loci were present in both populations. All but eight of the loci identified by Bayscan were also detected in the *F*_ST_ analysis.

### Environment variables and their association with loci

Annual precipitation and mean temperature exhibit a clear trend from north to south in Japan, and the snow depth differs greatly between the Japan Sea side and the Pacific Ocean side (Table 1). Cumulatively, the first three principal components explained 86.16% of the total variance in current environmental conditions (Table S3). Principal component 1 (PC1) was strongly related to the location of populations such as latitude, and PC2 and PC3 were explained mainly by temperature and precipitation in the dry season, respectively. The result of the PCA for the interglacial period (LIG) was very similar to that for current environmental conditions (Table S3). However, the results of the PCA for the last glacial maximum (LGM) were related to the temperature in the warmest season (PC2), precipitation in the dry season (PC2), average temperature in the dry season (PC3), and seasonal precipitation (PC3), although PC1 was highly related to latitude and seasonal temperature in the MIROC model.

In total, 38 loci were associated with environmental variables in at least one of the three climate condition scenarios: current, LIG, or LGM (Table 4). Of theses, 18 loci were detected in association with all three climatic conditions. With the exception of five loci, the detected loci in the current scenario were the same as those associated with the LIG, because the climatic conditions were similar. Eleven loci were only associated with the LGM.

**Table 4:** Strongly associated loci between the results of principal component analysis of environmental variables

Locus	Current_PC1	Current_PC2	Current_PC3	LIG_PC1	LIG_PC2	LIG_PC3	LGM_MIR_PC1	LGM_MIR_PC2	*F*_ST_	Linkage Group	Putative Function
gSNP06309	**			**			**		0.1459	—	Pinus taeda anonymous locus 0_14423_02 genomic sequence
gSNP11760	**			**			*		0.1482	7	Abscisic stress-ripening protein 3-like
gSNP06850	*	*			*		*	*	0.2450	10	No hit
estSNP00328	*			*			**		0.1922	8	udp-glucose pyrophosphorylase
estSNP00402	*			*			*		0.2338	1	50s ribosomal protein l9
estSNP01963	*			*			*		0.2194	—	50s ribosomal protein l9
estSNP02208	*			*			*		0.2486	6	ap2 erf domain-containing transcription factor
gSNP03082	*			*			*		0.1574	2	No hit
gSNP06599	*						*		0.1373	2	No hit
estSNP00454		*			*			*	0.1627	9	pi starvation-induced protein
estSNP01816		*			*				0.2397	—	Abscisic acid responsive elements-binding factor 2
estSNP02019		*			*			*	0.1627	—	pi starvation-induced protein
estSNP02622		*						*	0.1242	5	Exosome complex component csl4
estSNP03061		*			*			*	0.2224	—	Isochorismatase hydrolase family protein
gSNP01042		*			*				0.1415	10	Harpin-induced protein-like
gSNP01385		*			*				0.2228	—	No hit
gSNP04024		**	*		**	*		**	0.2556	11	R2R3-MYB transcription factor
estSNP00558			*			*		**	0.1539	2	DNA-binding storekeeper transcriptional regulator
estSNP01141			*			*		*	0.2413	—	e3 sumo-protein ligase mms21-like
estSNP01539			*			*	*	*	0.3186	—	Alpha-glucan-protein synthase
estSNP02553			*			*			0.1405	2	Hypothetical protein
gSNP07777			*						0.2796	5	No hit
gSNP09342			*			**		**	0.2689	10	No hit
estSNP02046			**			**		**	0.2365	2	Response to biotic stimulus
estSNP02882			**			**		**	0.2419	2	Metal-dependent phosphohydrolase hd domain-containing protein
estSNP00015								*	0.1612	3	structural constituent of
estSNP00110							*		0.1657	—	Phenylpropenal double-bond reductase
estSNP00178								**	0.1876	—	Ribosomal protein l13a
estSNP00338								*	0.1342	—	Proton gradient regulation 5
estSNP00625								**	0.1561	9	myb family transcription factor
estSNP00691						*			0.2334	—	Tannin-related r2r3 myb transcription partial
estSNP01232								*	0.1503	9	pdi-like protein
estSNP01913							*		0.2250	10	snf1-related protein kinase regulatory subunit beta-2-like
estSNP02482								*	0.2508	2	Proteolysis
estSNP03062						*		*	0.1772	2	DnaJ protein homolog
gSNP01330							*		0.2382	11	Adenosine 3-phospho 5-phosphosulfate
gSNP03195							*		0.1558	—	btb poz domain-containing protein at3g05675-like
gSNP11860							*		0.1571	4	3-ketoacyl-synthase

Loci detected by Principal component analysis (PCA) of environmental variables were also detected by both BayeScan (Bayes factor >2) and Lositan (*P* < 0.001), and their linkage group and putative function were detected by blastx search (E-value cut off ≤ 1 − 10^−3^). The “current” is the average climate data between 1950 and 2000, “LIG” is the average climate data of last interglacial between ∼120,000 and 140,000 years BP, and LGM is the average climate data of last glacial maximum at ∼21,000 years BP. *: p<0.01, **:p<0.001

All loci found to be related to an environmental variable were also identified as outliers in the Lositan, Arlequin, or BayeScan analyses. BLASTx searches identified putative functions for 37 of the 43 environment-associated loci.

### Linkage mapping of outlier loci

**Figure 5:**
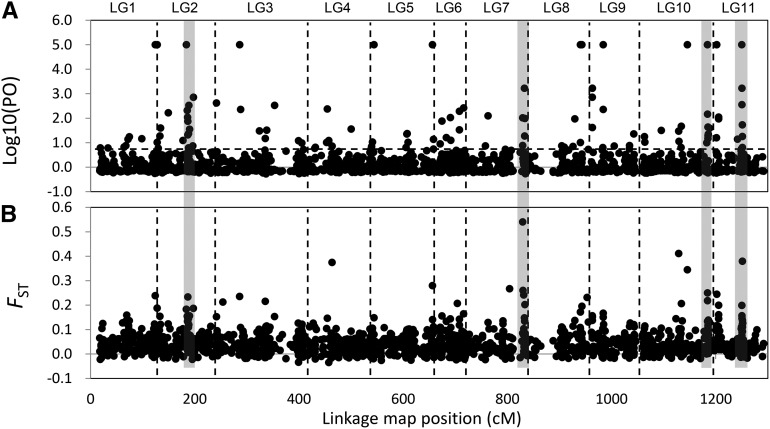
The distribution of markers identified as being potentially involved in local adaptation across the genome of *Cryptomeria japonica*. The figure shows plots of marker position (based on 1255 mapped SNPs or the other DNA markers; Moriguchi *et al.* 2012; Y. Moriguchi, T. Ujino-Ihara, K. Uchiyama, N. Futamura, S. Ueno, A. Matsumoto, and Y. Tsumura unpublished data) in cumulative centimorgans (cM) *vs.* (A) Bayes factor (log_10_), as determined by BayeScan analysis, and (B) *F*-statistic value, *F*_ST_. Vertical dashed lines demarcate the 11 linkage groups. The four gray zones indicate candidate regions associated with local adaptation.

In total, 208 loci were identified as outliers or loci associated with environmental variables for all populations; 101 of these loci were successfully mapped on the *C. japonica* linkage map, and their distribution was investigated (Figure 5) (Moriguchi *et al.* 2012). The numbers of mapped outlier loci in each linkage group varied substantially. For example, linkage groups (LGs) 4 and 5 contained only four loci, whereas LG2 contained 21. The observed distribution of loci deviated significantly from the Poisson distribution for all marker intervals considered (15, 20, 25, and 50 cM) and, therefore, was not random (*P* < 0.001). Thus, the outlier loci clearly formed clumps associated with specific linkage groups, particularly LG2, LG7, LG10, and LG11 (Figure 5). The outlier distribution was related to the *F*_ST_ distribution; regions with high *F*_ST_ values had high numbers of outlier loci.

### Linkage disequilibrium and the associated loci

**Figure 6:**
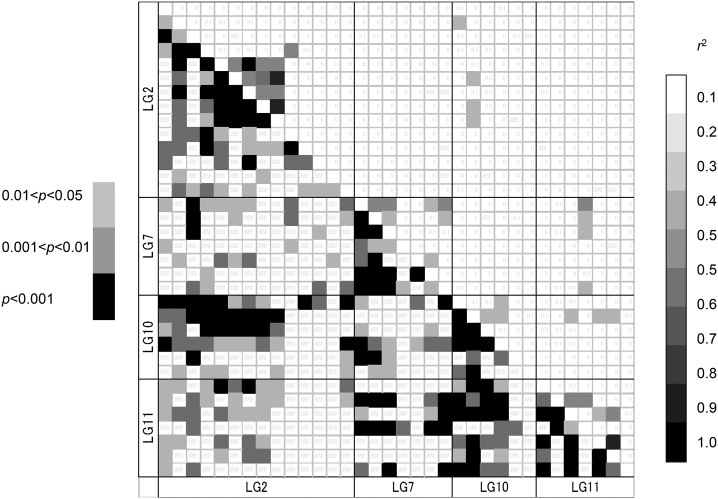
Heat maps of linkage disequilibrium (LD) values (*r*^2^) of the four regions, LG2, LG7, LG10, and LG11, and the *p* values.

Linkage disequilibrium (LD) was detected for 491,563 (6.37%, q < 0.1) of the 7,720,485 possible combinations of all 3930 loci with the false discovery rate of 0.1. However, the proportion of significant LD among the 208 outlier loci in all 14 populations was very high, 38.852% (q < 0.1) (Table 5). In particular, a significant LD was detected among the loci of the clumped regions, but inter-chromosomal LD was also detected, including for LG2 and LG10, LG10 and LG11 (Figure 6). The relatively higher *r*^2^ values (>0.1) between linkage groups were not frequent, which were gSNP09342 in LG10 and two loci in LG2, gSNP09342 in LG10 and three loci in LG11, gSNP09193 in LG11 and four loci in LG7, and estSNP02482 in LG2 and gSNP06850 in LG10. However, the proportion of significant LD was reduced to less than the half when we calculated the LD in each of the variety populations (ura-sugi and omote-sugi) and also the 13 populations excluding the YKU population (Table 5).

**Table 5:** The proportion of significant LD among the detected outlier loci and all loci

Investigated Population		n	loci	Average of *r*^2^	SD	*r*^2^ median	Proportion of Significant LD	
							*P* < 0.01	*q* < 0.1*
Outlier loci	All 14 populations	172	208	0.01729	0.04024	0.00786	0.27397	0.38852
	13 populations excluding Yakushima	158	205	0.01498	0.03920	0.00614	0.21019	0.29139
	Ura-sugi, 8 populations	101	202	0.01255	0.03895	0.00482	0.07950	0.06606
	Omote-sugi, 6 populations	71	208	0.02125	0.04449	0.00891	0.11994	0.13643
	Omote-sugi, 5 populations excluding Yakushima	54	205	0.01936	0.04164	0.00848	0.06432	0.04103
All loci	All 14 populations for all loci	172	3926	0.00646	0.01206	0.00290	0.07964	0.06367
	13 populations excluding Yakushima for all loci	158	3915	0.00684	0.01254	0.00308	0.07598	0.05733
	Ura-sugi, 8 populations for all loci	101	3875	0.01036	0.01668	0.00475	0.07195	0.05004
	Omote-sugi, 6 populations for all loci	71	3893	0.01493	0.02221	0.00702	0.07337	0.05136
	Omote-sugi, 5 populations excluding Yakushima for all loci	54	3868	0.01809	0.02628	0.00856	0.06933	0.04418

* False discovery rate (FDR 0.1).

We carefully recorded the allele frequency of each outlier loci in the four clumped regions and found that the genotypic frequency of most of loci is very different between populations of the two varieties (Table 6). The heterozygosity of populations of ura-sugi is lower in the three clumped regions than that of the omote-sugi populations for approximately 60% of the outlier loci. The seven outlier loci associated with LG7 formed a unique group that was specific to the YKU population, three loci of which (SNPg04215, SNPg07948, and SNPg06867) showed lower heterozygosity in all populations compared with the Yakushima population. Two loci (SNPg11760 and SNPg01044) exhibited very low heterozygosity only in the YKU population, and for the other two loci the allele frequency of the YKU population was different from that of the others.

**Table 6:** The outlier loci in four clumped regions of linkage map, their *F*_ST_, the heterozygosities, and the allele frequencies

Linkage Group	*: centiMorgan	Locus	*F*_ST_	Heterozygosity(*He*)			Allele Frequency of Allele		
				Ura-sugi Populations	Omote-sugi Populations	Yakushima Population	Ura-sugi Populations	Omote-sugi Populations	Yakushima Population
2	55.46	estSNP00091	0.1977	0.423	0.116	0.000	0.696	0.938	1.000
2	57.68	estSNP02482	0.2508	0.496	0.226	0.391	0.544	0.870	0.733
2	57.89	gSNP03082	0.1574	0.333	0.499	0.278	0.789	0.479	0.167
2	57.93	estSNP02390	0.1212	0.415	0.491	0.444	0.706	0.432	0.333
2	58.16	estSNP02046	0.2365	0.477	0.372	0.464	0.392	0.753	0.367
2	58.17	estSNP00558	0.1539	0.360	0.498	0.124	0.765	0.534	0.933
2	58.17	estSNP02882	0.2419	0.485	0.360	0.464	0.413	0.764	0.367
2	58.19	estSNP03062	0.1772	0.291	0.499	0.320	0.176	0.479	0.200
2	58.21	gSNP04589	0.1700	0.271	0.484	0.180	0.838	0.589	0.900
2	58.37	estSNP00649	0.1312	0.444	0.421	0.124	0.667	0.699	0.933
2	58.45	estSNP02061	0.1201	0.488	0.494	0.491	0.578	0.445	0.567
2	58.73	estSNP03044	0.1371	0.494	0.476	0.420	0.446	0.390	0.300
2	60.19	gSNP05191	0.1152	0.200	0.040	0.000	0.887	0.979	1.000
2	61.54	gSNP05463	0.1561	0.447	0.482	0.420	0.663	0.404	0.300
7	115.14	gSNP04215	0.5401	0.000	0.256	0.464	1.000	0.849	0.367
7	115.82	gSNP07948	0.2599	0.102	0.293	0.464	0.054	0.178	0.633
7	117.21	gSNP06867	0.2414	0.000	0.104	0.391	0.000	0.055	0.267
7	118.14	gSNP11760	0.1482	0.500	0.489	0.000	0.500	0.425	0.000
7	118.63	gSNP01044	0.1306	0.486	0.357	0.124	0.583	0.767	0.933
7	118.91	estSNP02454	0.1562	0.315	0.459	0.320	0.196	0.356	0.800
7	119.03	estSNP00146	0.1988	0.192	0.426	0.420	0.892	0.692	0.300
10	130.53	gSNP06850	0.2450	0.499	0.066	0.000	0.480	0.034	0.000
10	130.53	gSNP09342	0.2689	0.029	0.455	0.064	0.985	0.651	0.967
10	130.56	gSNP01042	0.1415	0.215	0.492	0.444	0.123	0.438	0.333
10	131.57	estSNP01913	0.2250	0.434	0.327	0.064	0.319	0.795	0.967
10	131.58	gSNP01835	0.1143	0.486	0.437	0.064	0.583	0.322	0.033
10	132.07	estSNP01295	0.1345	0.497	0.437	0.444	0.539	0.678	0.333
11	54.11	estSNP01481	0.1330	0.389	0.495	0.491	0.265	0.548	0.433
11	54.38	gSNP07270	0.1297	0.075	0.398	0.500	0.961	0.726	0.500
11	54.38	gSNP01805	0.1663	0.259	0.500	0.491	0.153	0.492	0.433
11	54.42	gSNP09193	0.3793	0.000	0.151	0.480	0.000	0.082	0.400
11	54.77	gSNP02822	0.1725	0.044	0.324	0.000	0.977	0.797	1.000
11	54.77	gSNP04480	0.1632	0.038	0.275	0.000	0.020	0.164	0.000
11	55.09	gSNP01330	0.2382	0.048	0.378	0.358	0.025	0.253	0.233

## Discussion

### Factors determining the genetic structure of *C. japonica*

The formation of the Japanese archipelago began approximately 17 million years ago, with the earliest incarnation of the Japan Sea forming approximately 7 million years ago (Taira 2001). A *Cryptomeria anglica* fossil dated to the later phase of the Miocene has been found in Japan and is a probable ancestor of *C. japonica*. Moreover, *C. japonica* fossils have been found to date back to the Pleiocene, thus postdating the formation of the Japanese archipelago (Uemura 1981). *C. japonica* has therefore adapted to climatic differences over extended periods of time while shifting its distribution in the face of global climate changes, including several glacial and interglacial periods.

The estimated current potential natural distributions are much wider than the observed natural distribution (Kimura *et al.* in press) (Figure S2), which means that interspecific competition, for example, competition with evergreen tree species in western Japan (Takahara 1998), and historical human disturbance associated with timber extraction strongly influenced the distribution we see today. The estimated natural distribution using the MIROC model suggests small northern refugia during the LGM (Figure S2).

The network tree and the structure results clearly divided the 14 study populations into two variety-based groups, as have previous studies (Tsumura *et al.* 2007, 2012). The ONN population belongs to the ura-sugi and is close to the AZJ population; however, the long branch-length in the network tree suggests a bottleneck and isolation. In fact, a limited number of individuals survive in the ONN population at relatively higher elevations, the sole natural population on Kyushu Island. We selected the 14 populations investigated to cover the whole natural distribution area, based on geographical location. The ONN population is closer to the Pacific Ocean side of the country, and thus this population was included as an omote-sugi population. Its phenotype, especially for the needles, is intermediate between the ura-sugi and omote-sugi varieties (Nakao *et al.* 1986). The AST, KWZ, and YKU populations were mostly fixed within the ura-sugi cluster, representing the main refugia during the LGM; however, the ISN, SNG, and KCH populations have some associations with the ura-sugi cluster, probably because they experienced introgression from ura-sugi populations during population expansion after LGM. From fossil pollen data, Takahara (1998) suggested that, in western Japan, especially on Sikoku Island and probably Kyushu Island, the expansion of natural forests of *C. japonica* along the Pacific Ocean side may have been much slower than along the Japan Sea side because of the rapid expansion of evergreen broadleaf forest during the postglacial period. This may be one reason why the origin of the sole natural population on Kyushu Island, the ONN population, is on the Japan Sea side. The YKU population also has a long branch-length in the network tree, suggesting long-term isolation. The YKU population is the most southerly population and an extensive natural population has been maintained for a long time because it occurs on a small isolated island with steep mountains, offering a preferred habitat for this species (Tsumura and Ohba 1993). It is thought that this island became isolated from Kyushu Island during the LGM. However, the vegetation was severely damaged by the eruption of Kikai caldera approximately 40 km north from Yakushima Island, 7300 years ago, and it is likely that only the vegetation of the southern coastal area survived this event (Geshi 2009). This reduction in population size with the isolation from Kyushu Island may explain the characteristics of the YKU population. The Neighbor-net output suggests some degree of reticulation between the two varieties; this suggestion is also supported by the admixture revealed by the STRUCTURE analysis.

According to fossil pollen data (Tsukada 1983, 1988), the refugial areas of this species during the last glacial period (approximately 18,000 years ago) were along Wakasa Bay to Oki Island (ura-sugi variety), on the Izu Peninsula, Yakushima Island, and (probably) the southern Kii Peninsula and Shikoku Island (omote-sugi variety) (Figure 1). Before the LGM, the two varieties had already diverged (Kimura *et al.* in press) and the genetic structure developed further as a result of global climate change, for example, during the glacial and interglacial periods. These northern populations are thought to have established approximately 6000 years ago by migration from refugia, based on inferences from fossil pollen data (Tsukada 1986). If so, then the genetic diversity of the most northerly marginal forests was reduced by bottleneck effects. However, a few populations in northern areas have relatively high genetic diversity compared with those of the other northern populations, suggesting that some small populations may have survived in the northern area even during the last glacial period (Takahara 1998). *C. japonica* trees can sometimes survive severe climate conditions, such as low temperatures and heavy snowfalls, which are common at high elevations. For example, at 2050 m (Taira *et al.* 1993), most seedlings cannot survive but a few trees are able to regenerate by layering, and this can allow them to persist for a long time (Taira *et al.* 1997; Moriguchi *et al.* 2001). Such small populations may have survived in refugia in northern Japan during glacial periods; this suggestion is supported by the structure result for K = 4.

Genetic differentiation between populations was small (average *F*_ST_ = 0.0506), as found in previous studies using SSR and CAPS (Takahashi *et al.* 2005; Tsumura *et al.* 2007). The distribution has shifted because of climate change, and small refugia in marginal areas may have supported founder populations; thereafter, the genetic differentiation could be expected to have been large because of the founder effect. However, the long life-cycle of tree species may have provided sufficient pollen flow between populations, so that the founder effect was limited and the genetic differentiation was suppressed (Austerlitz *et al.* 2000). However, even the limited genetic differentiation is associated with some important loci linked to adaptation to the different environments experienced during the winter on the Japan Sea side and Pacific Ocean side of the country.

### How did natural selection influence to the genetic structure?

The natural *C. japonica* forest has a wide distribution, from 30°N, 130°E to 40°N, 140°E (Figure 1). There is heavy snowfall on the Japan Sea side of the country in the winter, but the Pacific Ocean side is quite dry in the winter, and the annual precipitation is much higher in the southern part of Japan than elsewhere in the country. Thus, there are significant differences in the environmental factors affecting the different regions examined in this work, especially in terms of annual precipitation and the maximum depth of fallen snow (Table 1). There is a cline of environmental variables including precipitation, temperature, and snowfall across the Japanese archipelago from the northeast to southwest. The key environmental difference between the Japan Sea and Pacific Ocean sides of Japan is snowfall. This difference in environments exerts an important selection pressure. The results of our PCA indicate that the current natural distribution is positively linked to winter precipitation; the natural distribution during the LGM was also positively linked to the maximum and mean temperatures during the warmest period of the year. *C. japonica* has adapted well to temperate climates and moist regions. Normally, it can grow in areas with minimum temperatures during the coldest month of approximately −7° to 2°, maximum temperatures during the warmest month of 22° to 28°, and effective annual precipitation exceeding 1500 mm. The current distribution seems to be strongly restricted by precipitation, especially in winter, which is the coldest and driest season. The LGM distribution was probably limited by maximum temperature and also precipitation. The results of the species distribution model support this conclusion (Figure S2).

Two-hundred eight outlier loci were detected using two different methods—Lositan and Bayscan (Table S1). Of these, 43 loci were also associated with environmental variables (Table 4). The detected outlier loci that were associated with environmental variables represent only 1.1% of all analyzed loci; this is a similar proportion to that reported in previous studies (2.7% and 1.4%) (Tsumura *et al.* 2007, 2012). Eckert *et al.* (2010) identified 22 candidate loci (BF log_10_ > 2.0) associated with climatic variables from a total of 1730 loci in 682 loblolly pine tree samples covering the full range of the species. Prunier *et al.* (2011) also identified 10 candidate loci above the 99% C.I. in black spruce. The proportion of detected loci (1.3% and 1.7%, respectively) associated with the climatic variables was very similar to that found in the current study. The detected outlier loci among the current, last interglacial (LIG), and LGM climate variables were very similar to each other, especially the first two because of their similar climatic conditions. The 18 outlier loci found to be associated with conditions during the LGM may be linked to low temperature and/or limited precipitation. In fact, five of these genes are associated with stress responses. The functions of 41 of these 208 outlier loci are associated with stress responses, and nine of the 34 outlier loci in the four clumped regions of the genome are also associated with stress responses. Of these, LG2 includes five stress response genes. However, the putative functions of these outlier loci identified by the Blastx search could not be explained by the adaptive mechanisms. This indicates that these regions might be experiencing selection. However, it is important to account for hitchhiking effects properly when making suggestions of this kind, because it is possible that the genes identified may simply be closely linked to the true adaptive gene or genome region.

The mapping result clearly showed the clumped distribution of the outlier loci on the linkage map. We found four clumped regions in this study (Table 6), of which LG2 and LG11 had already been detected in a previous study (Tsumura *et al.* 2012). The four regions detected ranged from 1 cM (LG11) to 6 cM (LG2) on the genetic map, and 6 to 14 outlier loci were mapped in these regions. Their *F*_ST_ was more than 0.11 (average 0.19), although the average of all 3930 loci was low (*F*_ST_ = 0.0506). These high *F*_ST_ values strongly suggest a hitchhiking effect. These regions could be considered to represent a “genomic island of divergence” (Nosil *et al.* 2009). The detected four regions might be merely controlled by four adaptive loci, and thus their surrounding loci might be detected by the hitchhiking effect of the true adaptive locus. Scotti-Saintagne *et al.* (2004) also identified some important regions of genomes associated with adaptation using both outlier detection and QTL mapping methods. The proportion of significant LD among the 208 outlier loci was quite high (27.40%, *P* < 0.01) because not only the four regions but also the other outlier loci are sometimes tightly linked to each other. Such important genes sometimes comprise clusters on a genome, for example, defense-related genes (Boyko *et al.* 2002), which are important complex traits (Yamamoto *et al.* 2009; Messmer *et al.* 2009). The LD in conifers is thought to decay rapidly for several thousand bps (Neale and Savolainen 2004), but most data have been collected from coding genes. A recent study interestingly revealed LD in a noncoding region in *C. japonica* that does not decay at least for several hundred kbp (Moritsuka *et al.* 2012). Generally, the LD is thought to be highly structured into discrete blocks of sequences separated by hot spots of recombination (Goldstein 2001; Kim *et al.* 2007); therefore, the discovery of a noncoding LD may not be surprising. In this study, we investigated SNPs within coding regions; therefore, the detected LD between the outlier loci may provide evidence of selection. The proportion of significant LD among the outlier loci in all 14 of our *C. japonica* populations was quite high, but the significant LD in populations for each variety was low (Table 5), suggesting that selection between two varieties might have occurred. Interchromosomal LD between two varieties is also strong evidence for selection affecting them differently. The average *r*^2^ value of the outlier loci is higher than that of all loci (Figure 6, Table 5). The Yakushima (YKU) population has an effect on this high LD because of the relatively long time it has been isolated from the main population. The higher average *r*^2^ value between populations of omote-sugi is related their distribution: the populations are scattered and relatively small compared with those of ura-sugi. The average *r*^2^ value for the outlier loci of omote-sugi populations is higher than that for all 14 populations; however, the proportion of significant LD within the omote-sugi variety is low, which means some specific combinations of loci between omote-sugi populations have quite high *r*^2^ values. The average *r*^2^ value of the outlier loci is not particularly high, but the proportion of significant LD is quite high compared with that of all loci, which means that most of these outlier loci could have been detected because of the hitchhiking effect. The relatively higher *r*^2^ values (>0.1) between linkage groups only applied to 14 combinations of loci. The allele frequencies of these loci between populations of two varieties were significantly different; these were gSNP09342 in LG10 and two loci (estSNP03062 and gSNP04589) in LG2 and gSNP09342 in LG10 and three loci (gSNP01805, gSNP02822, and gSNP04480) in LG11. In addition, there were significant differences between gSNP09193 in LG11 and four loci (gSNP04215, gSNP07948, gSNP06867, and estSNP00146) in LG7; in this case, the allele frequencies were distinctive in that only the YKU population was highly heterozygote. Therefore, the LDs between these interchromosomal loci were relatively higher; however, it is still unclear whether such interchromosomal LD is created by selection or chance. Interchromosomal LD was detected in cultivated tomato, but this LD was caused by artificial selection (Robbins *et al.* 2011). Evolutionary mechanisms, such as co-adapted complexes of genes, might affect such LD (Dobzhansky 1970). If the detected outlier loci include many rare variants (Dickson *et al.* 2010), then the possibility of a falsely significant LD is increased. However, only 12.98% of outlier SNPs have MAF (minor allele frequency) < 0.1 compared with 16.84% of all SNPs with MAF < 0.1. An elevated MAF distribution in the outlier SNPs suggests these might be older SNPs rather than recently selected alleles. Identical genes have been mapped on a linkage map as different loci due to genotyping errors, and thus we carefully checked the putative function of each mapped locus by a Blastx search. In fact, the loci, gSNP01044 and estSNP02454, were tightly linked on LG7 and are probably splicing variants of the same coding region with the same function based on the sequence data of BAC clones including the SNP; however, the other tightly linked loci did not have identical functions, which means that the mapped loci associated with the four regions are independent genes and specific adaptive haplotypes that could be maintained in each variety.

The LG7 region is characteristic of the YKU population and, therefore, the region may be the result of adaptation to a specific environment, such as the oligotrophic granite soil or the abundant precipitation (>4000 mm). The other three regions—LG2, LG10, and LG11—are related to each other. In particular, the four loci, gSNP06850, gSNP09342, gSNP01042, and estSNP01913, on LG10 have significant interchromosomal LD with loci of LG2 and LG11, for which the *r*^2^ values are relatively high. These three LD regions may be associated with differences in adaptation between the two varieties.

The selective divergence associated with the clumped regions could be older, with the selective advantage of local alleles preventing establishment of alleles entering populations by gene flow (Maynard Smith and Haigh 1974; Oleksyk *et al.* 2010). Because the heterozygosity in populations of ura-sugi is lower than that of omote-sugi populations, especially for the clumped region of LG11, most of the ura-sugi loci are fixed homozygotes (Table 6). The four regions probably contain important genes associated with adaptation to different environments and/or morphological traits that differ between the two varieties.

One adaptive mechanism of such a genome region is thought to be associated with genome inversion, because inversions can suppress recombination and thus protect adaptive alleles from gene flow (Kirkpatrick and Barton 2006; Machado *et al.* 2007; Fang *et al.* 2012). An inversion that captures two or more alleles that are adapted to the local environmental conditions has a selective advantage that can cause it to spread (Kirkpatrick 2010). The yellow monkeyflower provides a good example of local adaptation occurring in two distinct ecotypes. The inversion polymorphism has been found to affect adaptive flowering time divergence and other morphological traits in all replicated crosses between four pairs of annual and perennial populations in *Mimulus guttatus* (Lowry and Willis 2010). In our study, the basic linkage map was constructed for the family of parent trees belonging to the omote-sugi variety (Moriguchi *et al.* 2012). We are currently unable to determine whether the inversion polymorphism is associated with adaptation in *C. japonica*; therefore, it is better to use the ura-sugi variety parents to investigate the inversion. If the inversion is detected, then we will prepare the F_2_ populations between the parents of the omote-sugi and ura-sugi varieties for reciprocal transplanting to examine the association between adaptive traits and the inversion polymorphism.

The detected outlier loci of the four regions with high LD are strong candidates for involvement in local adaptation. It is necessary to confirm whether these regions include genuine adaptive genes, comparing the sequences of these genes and surrounding regions using a BAC library to investigate the flanking regions. The identification of outlier loci with high significance levels is essential for conservation purposes and will be required for future work regarding molecular breeding.

## Supplementary Material

Supporting Information
