# Supplementary material for: Genetic Differentiation and Evolutionary Adaptation in Cryptomeria japonica
Source: G3 (Bethesda). 2014 Oct 14;4(12):2389–402. doi: 10.1534/g3.114.013896 (PMC4267934; doi:10.1534/g3.114.013896)
Supplement: Supporting Information [file supp_4_12_2389__index.html]

Genetic Differentiation and Evolutionary Adaptation in Cryptomeria japonica — Supporting Information 

# Genetic Differentiation and Evolutionary Adaptation in *Cryptomeria japonica*

## Supporting Information for Tsumura *et al.*, 2014

**Files in this Data Supplement:**

- Supporting Information - Figures S1-S2 and Tables S1-S3 (PDF, 188 KB)
- Figure S1 - Venn diagram of outlier loci detected by Lositan, Arlequin and BayeScan. (PDF, 103 KB)
- Figure S2 - The estimated current and potential LGM natural distributions of *C. japonica*. (PDF, 172 KB)
- Table S1 - Outlier loci detected by Lositan and Bayscan (P<0.01). (.xlsx, 31 KB)
- Table S2 - Outlier loci detected in each variety group. (.xlsx, 20 KB)
- Table S3 - The results of Principal Component Analysis (PCA) of environmental variables. (.xlsx, 12 KB)
