# Supplementary material for: Genetic Differentiation and Evolutionary Adaptation in Cryptomeria japonica
Source: G3 (Bethesda). 2014 Oct 14;4(12):2389–402. doi: 10.1534/g3.114.013896 (PMC4267934; doi:10.1534/g3.114.013896)
Supplement: Supporting Information [file supp_g3.114.013896_013896SI.pdf]

G3: Genes | Genomes | Genetics

Genetic differentiation and evolutionary adaptation in *Cryptomeria japonica*

Yoshihiko Tsumura[1], Kentaro Uchiyama[1], Yoshinari Moriguchi[2], Megumi K. Kimura[1,3], Saneyoshi Ueno[1] and Tokuko Ujino-Ihara[1]

1: Department of Forest Genetics, Forestry and Forest Products Research Institute, Tsukuba Ibaraki 305-8687, Japan

2: Graduate School of Science and Technology, Niigata University, 8050, Igarashi 2-Nocho, Nishi-ku Niigata 950-2181, Japan.

3: Present address: Forest Tree Breeding Center, Forestry and Forest Products Research Institute, 3809-1, Ishi, Juo, Hitachi 319-1301, Japan

Address for corresponding author;

Yoshihiko Tsumura

Department of Forest Genetics, Forestry and Forest Products Research Institute, Tsukuba, Ibaraki 305-8687, Japan

Phone +81-29-829-8260

Fax +81-29-874-3720

E-mail; ytsumu@ffpri.affrc.go.jp

DOI: 10.1534/g3.114.013896

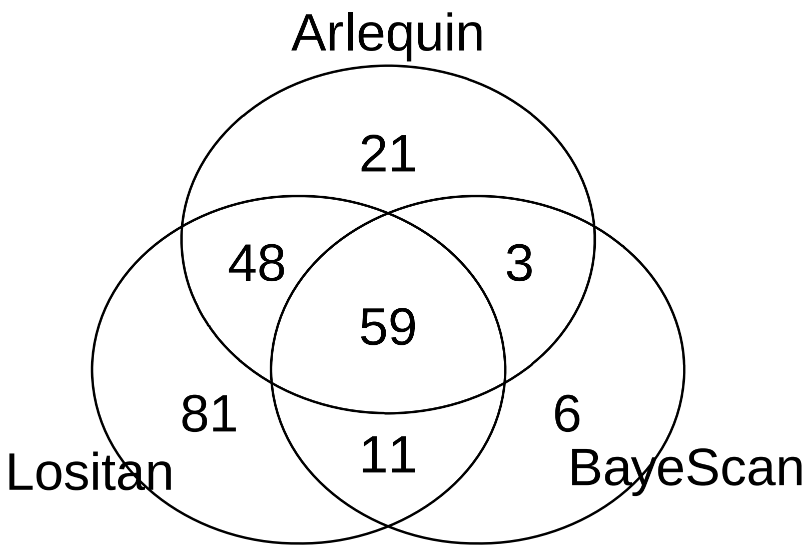

Figure S1 Venn diagram of outlier loci detected by Lositan, Arlequin and BayeScan.

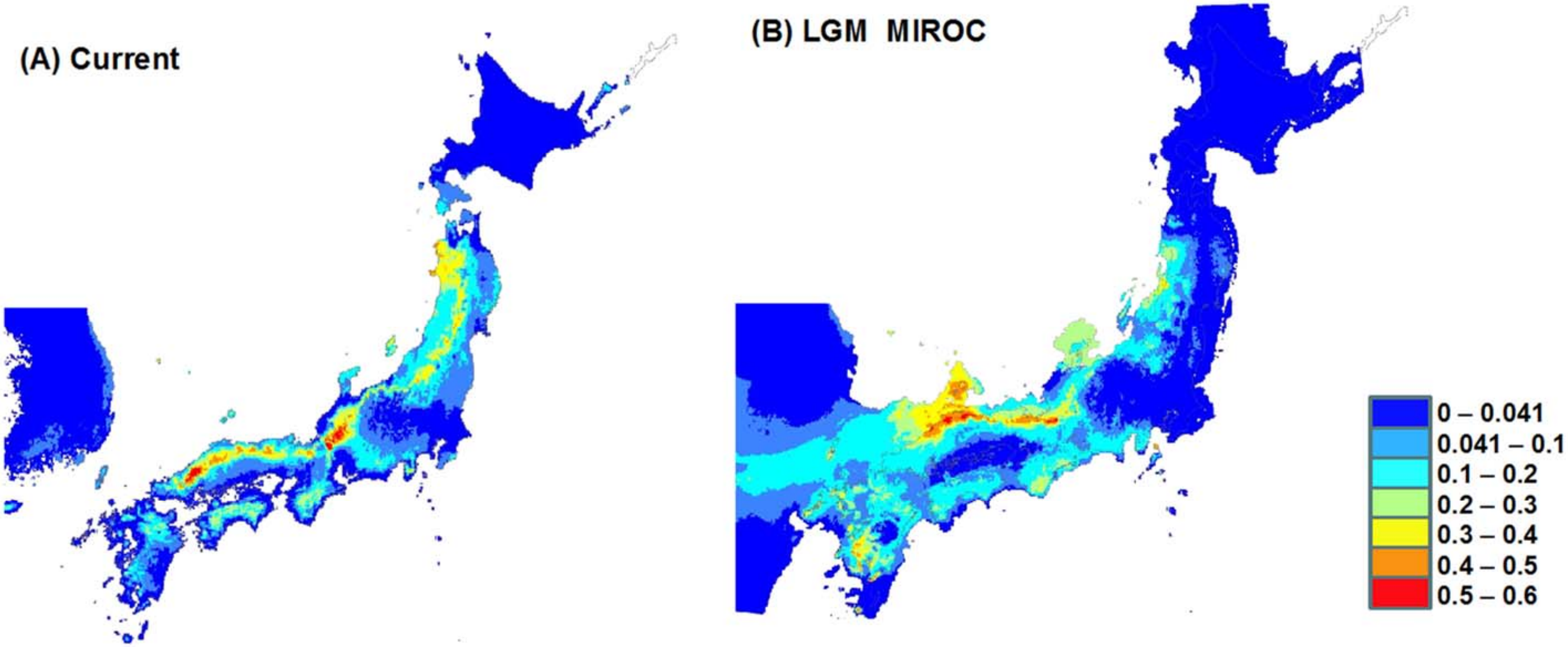

Figure S2 The estimated current and potential LGM natural distributions of *C. japonica*.

Tables S1-S3

Available for download as Excel files at http://www.g3journal.org/lookup/suppl/doi:10.1534/g3.114.013896/-/DC1

Table S1 Outlier loci detected by Lositan and Bayscan (P<0.01).

Table S2 Outlier loci detected in each variety group.

Table S3 The results of Principal Component Analysis (PCA) of environmental variables.
