# Supplementary figures and images for: Genetic Differentiation and Evolutionary Adaptation in Cryptomeria japonica
Source: G3 (Bethesda). 2014 Oct 14;4(12):2389–402. doi: 10.1534/g3.114.013896 (PMC4267934; doi:10.1534/g3.114.013896)

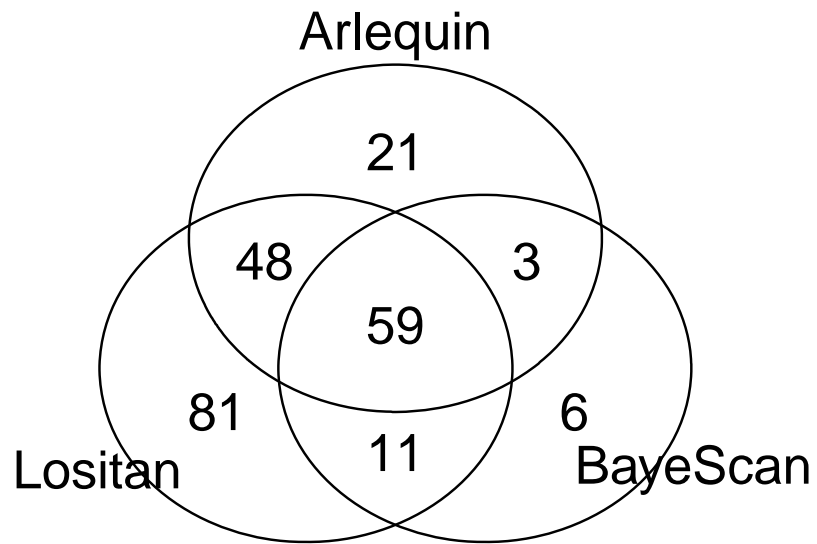

**Figure S1** Venn diagram of outlier loci detected by Lositan, Arlequin and BayeScan.

Supplement: Supporting Information [file supp_g3.114.013896_FigureS1.pdf]

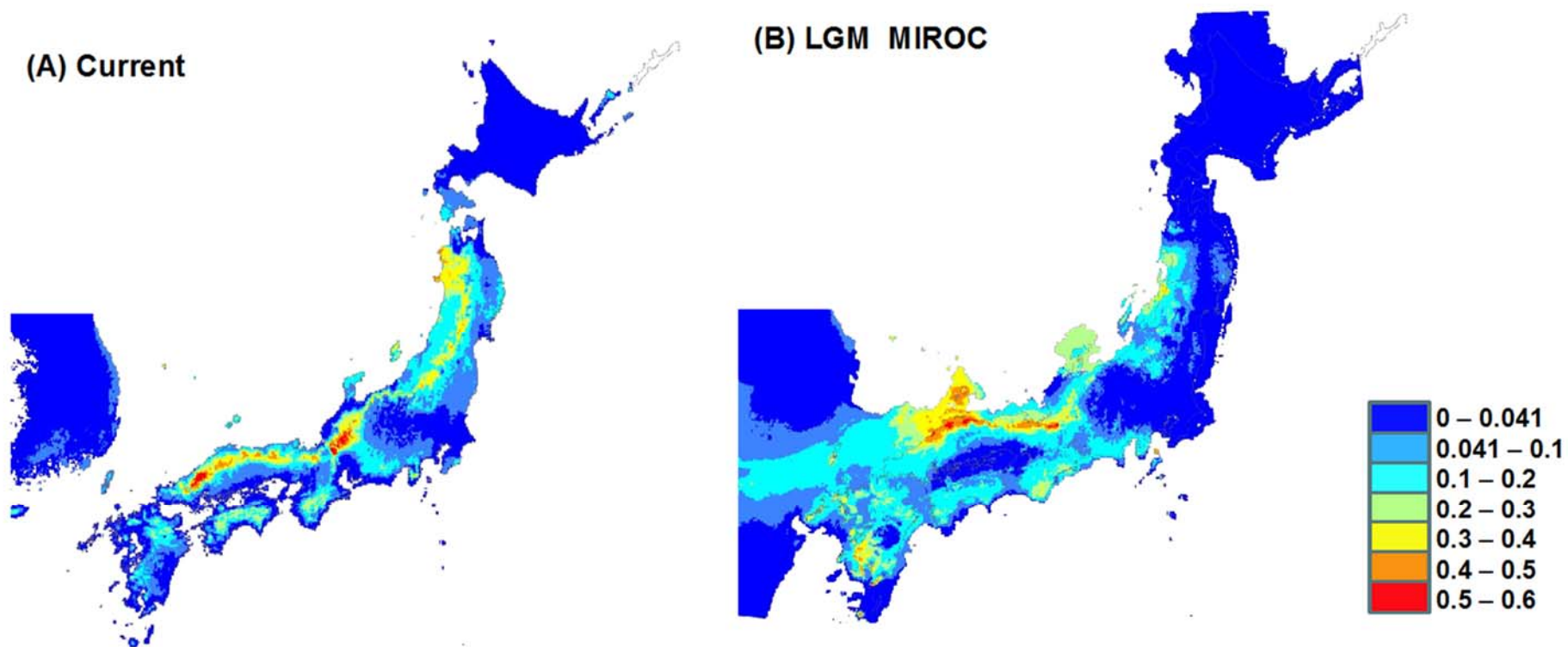

**Figure S2** The estimated current and potential LGM natural distributions of *C. japonica*.

Supplement: Supporting Information [file supp_g3.114.013896_FigureS2.pdf]
